# YKL-40 changes are not detected in post-mortem brain of patients with Alzheimer’s disease and frontotemporal lobar degeneration

**DOI:** 10.1186/s13195-022-01039-y

**Published:** 2022-07-25

**Authors:** Yanaika S. Hok-A-Hin, Jeroen J. M. Hoozemans, William T. Hu, Dorine Wouters, Jennifer C. Howell, Alberto Rábano, Wiesje M. van der Flier, Yolande A. L. Pijnenburg, Charlotte E. Teunissen, Marta del Campo

**Affiliations:** 1 Neurochemistry Laboratory, Clinical Chemistry department, Amsterdam Neuroscience, VU University Medical Center, Amsterdam UMC, Amsterdam, The Netherlands; 2 Department of Pathology, Amsterdam Neuroscience, VU University Medical Center, Amsterdam UMC, Amsterdam, The Netherlands; 3 Department of Neurology, Center for Neurodegenerative Diseases Research, Alzheimer’s Disease Research Center, Emory University School of Medicine, Atlanta, USA; 4 CIEN Tissue Bank, Alzheimer’s Centre Reina Sofía-CIEN Foundation, Madrid, Spain; 5 Alzheimer Centre Amsterdam, Department of Neurology, Amsterdam Neuroscience, VU University Medical Centers, Amsterdam, The Netherlands; 6 Department of Epidemiology and Data Science, VU University Medical Centers, Amsterdam, The Netherlands; 7 Departamento de Ciencias Farmacéuticas y de la Salud, Facultad de Farmacia, Universidad San Pablo-CEU, CEU Universities, Madrid, Spain

**Keywords:** YKL-40, Chitinase 3-like I, Alzheimer’s disease, Frontotemporal lobar degeneration, Neuroinflammation, Brain

## Abstract

**Background:**

YKL-40 (Chitinase 3-like I) is increased in CSF of Alzheimer’s disease (AD) and frontotemporal lobar degeneration (FTLD) patients and is therefore considered a potential neuroinflammatory biomarker. Whether changed YKL-40 levels in the CSF reflect dysregulation of YKL-40 in the brain is not completely understood yet. We aimed to extensively analyze YKL-40 levels in the brain of AD and different FTLD pathological subtypes. The direct relationship between YKL-40 levels in post-mortem brain and ante-mortem CSF was examined in a small set of paired brain-CSF samples.

**Method:**

YKL-40 was analyzed in post-mortem temporal and frontal cortex of non-demented controls and patients with AD and FTLD (including FTLD-Tau and FTLD-TDP) pathology by immunohistochemistry (temporal cortex: 51 controls and 56 AD and frontal cortex: 7 controls and 24 FTLD patients), western blot (frontal cortex: 14 controls, 5 AD and 67 FTLD patients), or ELISA (temporal cortex: 11 controls and 7 AD and frontal cortex: 14 controls, 5 AD and 67 FTLD patients). YKL-40 levels were also measured in paired post-mortem brain and ante-mortem CSF samples from dementia patients (*n* = 9, time-interval collection: 1.4 years) by ELISA.

**Results:**

We observed that YKL-40 post-mortem brain levels were similar between AD, FTLD, and controls as shown by immunohistochemistry, western blot, and ELISA. Interestingly, strong YKL-40 immunoreactivity was observed in AD cases with cerebral amyloid angiopathy (CAA; *n* = 6). In paired CSF-brain samples, YKL-40 concentration was 8-times higher in CSF compared to brain.

**Conclusion:**

Our data suggest that CSF YKL-40 changes may not reflect YKL-40 changes within AD and FTLD pathological brain areas. The YKL-40 reactivity associated with classical CAA hallmarks indicates a possible relationship between YKL-40, neuroinflammation, and vascular pathology.

**Supplementary Information:**

The online version contains supplementary material available at 10.1186/s13195-022-01039-y.

## Background

Compelling evidence from clinical and preclinical studies indicate that neuroinflammatory processes are relevant mediators in the development of different neurodegenerative dementias such as Alzheimer’s disease (AD) and frontotemporal lobar degeneration (FTLD) [1, 2]. Chronic activation of glial cells has shown to exacerbate AD and FTLD pathology and phenotypes [3–5] and modulate disease progression [2, 6, 7]. Moreover, causal and susceptibility genes associated with AD or FTLD (e.g., *TREM-2*, *CD33, GRN*) are related to neuroinflammation [8, 9]. Biomarkers that accurately reflect such neuroinflammatory processes *in vivo* could aid in the development of tools tracking disease progression and disease-modifying therapies [10, 11].

YKL-40 (or chitinase-3-like protein 1) is a glycoprotein involved in different mechanisms such as extracellular matrix remodeling [12, 13] or endothelial dysfunction [14]. It is also involved in the innate immune system [15] and has shown to be secreted by activated macrophages [16]. Several independent studies have shown that YKL-40 levels are increased in the cerebrospinal fluid (CSF) of AD [17–25] and FTLD patients [20, 21, 23, 26–31]. In brain, YKL-40 immunoreactivity has mainly been detected in astrocytes from controls and different tauopathies (AD and FTLD-Tau), whereby it correlated positively with tau pathology [22, 32] and surrounded amyloid-beta plaques in AD [17, 22, 32]. YKL-40 is also present in brain tissue from different neurological conditions (e.g., multiple sclerosis; MS, Creutzfeldt-Jakob disease; CJD, and bacterial meningitis) [22, 33, 34]. In MS, YKL-40 was detected in both astrocytes and microglial cells which correlated to the high inflammatory state of the disease [34]. These CSF and post-mortem brain data suggest that YKL-40 is a promising biomarker reflecting the neuroinflammatory process in different neurodegenerative dementias.

Several studies have analyzed the cellular location YKL-40 in the brain of AD, FTLD, and MS cases [17, 22, 32, 34]. However, few studies have analyzed YKL-40 levels using semi-quantitative approaches [22, 32], which revealed that the number of YKL-40 positive astrocytes was overall relatively limited, representing less than 10% of all GFAP-positive astrocytes [32]. Also, the correlation between YKL-40 levels in brain and CSF is rather unknown. Here, we aimed to further understand YKL-40 expression in brain and its relation with ante-mortem CSF. Thus, we analyzed YKL-40 levels in a large sample set of post-mortem human brain of non-demented controls and dementia cases with different underlying pathologies (AD, FTLD-Tau, and FTLD-TDP). Furthermore, we also examined the association between the levels of YKL-40 in post-mortem brain and ante-mortem CSF in a small set of paired brain-CSF samples from dementia patients.

## Methods

### Human samples

#### Post-mortem brain tissue

Temporal cortex of AD patients (*n* = 56) and non-demented controls (NDC, *n* = 51) were obtained from the Netherlands Brain Bank (Amsterdam). Six of these AD cases had severe cerebral amyloid angiopathy (CAA) type-1 and no or low numbers of parenchymal amyloid deposits [35]. Frontal cortex of cases with AD (*n* = 4) and FTLD (*n* = 67) pathology and NDC (*n* = 13) were obtained from the Netherlands Brain Bank (FTLD *n* = 21 and NDC = 5), CIEN Foundation Brain bank (AD *n* = 4, FTLD *n* = 23, and NDC *n* = 4; BT-CIEN, Madrid, Spain) and Emory University (FTLD *n* = 22 and NDC *n* = 4). The FTLD group included cases with pathological diagnosis of FTLD-Tau (*n* = 33) and FTLD-TDP (*n* = 34). FTLD-Tau group consisted of different forms of tauopathies such as progressive supranuclear palsy (PSP, *n* = 11), corticobasal degeneration (CBD, *n* = 3), and Pick’s disease (PiD, *n* = 3). Familial cases with *MAPT* mutation were also included (*n* = 16) [36]. The FTLD-TDP group also included familial cases with *C9orf72* (*n* = 7) or *GRN* (*n* = 6) mutations [37, 38]. Pathological examination was performed following consensus guidelines described previously [39–43]. As far as stated in the autopsy report, controls with no clinically relevant inflammatory-related diseases were excluded. Written informed consent for brain autopsy and the use of medical records for research purposes was given by all donors or their next of kin. An overview of patient details including age, sex, and post-mortem delay is summarized in Table 1. Additional specific information per case is provided in Supplementary Table 1.

**Table 1:** Demographic data of post-mortem brain samples

	*Temporal cortex*		*Frontal cortex*		
Technology	CON	AD	CON	AD	FTLD
IHC					
*n* (M/F)	51 (19/32)	56 (16/40)	7 (4/3)	-	24 (11/13)
Age, years (mean ± SD)	79 (12)	81 (10)	70 (10)	-	68 (10)
PMD, hours (mean ± SD)	6 (3)	5 (1)	5 (4)	-	6 (2)
FTLD subgroups					
FTLD-Tau					*4 PSP, 1 CBD, 6 PiD, 8 MAPT*
FTLD-TDP					*5 TDP pathology*
WB/ELISA^$^					
*n* (M/F)	11 (2/9)	7 (4/3)	14 (8/6)	5 (5/0)	67 (32/35)
Age, years (mean ± SD)	72 (12)	76 (8)	67 (9) ^A^	80 (11)^B, C^	67 (9)^A^
PMD, hours (mean ± SD)	6 (3)	6 (1)	7 (3)	5 (1)	8 (6)
FTLD Subgroups					
FTLD-Tau					*11 PSP, 3 CBD, 3 PiD, 16 MAPT*
FTLD-TDP					*21 TDP pathology, 7 C9orf72, 6 GRN*

*CON*, non-demented controls; *AD*, Alzheimer’s disease; *FTLD*, frontotemporal lobar degeneration; *TDP*, TAR DNA-binding protein; *PiD*, Pick disease; *PSP*, progressive supranuclear palsy; *CBD*, corticobasal degeneration; *n*, number of cases; *M*, male; *F*, female; *PMD*, post-mortem delay; *IHC*, immunohistochemistry; *WB*, western blot; *ELISA*, enzyme-linked immuno sorbent assay

*P*<0.05 from (A) AD, (B) CON, or (C) FTLD group

$ indicates Con and AD temporal cortex tissue that was analyzed only by ELISA due to limited availability

For immunohistochemistry, paraffin-embedded brain tissue was obtained. Sections of 5-μm thick were mounted on tissue slides (Superfrost® plus, Menzel Glaser, Braunschweig, Germany) and dried overnight at 37°C. For enzyme-linked immunosorbent assay (ELISA) and western blot analysis, frozen brain tissue blocks were provided and homogenized using Tissue Protein Extraction Reagent (T-PER, 0.1 g/mL, Thermo Scientific, Waltham, USA) containing EDTA-free protease inhibitor cocktail (1:25, Roche, Basel, Switzerland) and phosphatase inhibitor (1:10, Roche, Basel, Switzerland). Tissue homogenates were centrifuged at 10.000 *g* at 4°C for 5 min. Thereafter, total protein content for the supernatant was measured using the Pierce™ BCA Protein Assay Kit (Thermo Scientific, Waltham, USA) following the manufacturer's instructions. Brain lysates were aliquoted and stored at -80°C until further analysis.

#### Paired ante-mortem CSF sand post-mortem brain samples

Paired ante-mortem CSF and post-mortem frontal cortex samples (*n* = 9) were obtained from Emory University which included different types of autopsy-confirmed dementias (FTLD-Tau = 3, FTLD with amyotrophic lateral sclerosis; FTLD-ALS = 5, and dementia with Lewy body; DLB = 1). FTLD-ALS associates with TDP-43 pathology [44]. Neuropathology for TDP and DLB was determined following international guidelines [40, 45]. CSF material was obtained by standard lumbar puncture and collected in polypropylene (PP) tubes. CSF was aliquoted and stored at −80°C prior to analysis. CSF total protein content was measured using the Pierce™ BCA Protein Assay Kit. Informed consent was provided from subjects or their next of kin. The demographic data of cases included in this study are summarized in Supplementary Table 2.

### Immunohistochemistry

Brain sections (*n* = 134) were deparaffinized and immersed for 30 min in 0.3% H_2_0_2_ to quench endogenous peroxide activity. Antigen retrieval was performed using sodium citrate buffer (10 mmol/L, pH 6.0) for 20 min. Sections were incubated overnight at 4°C in a humid environment with Goat anti-human Chitinase 3-like 1 Antibody (1:50, Cat. No AF2599, R&D Systems, Minneapolis, USA) in antibody diluent (Immunologic, Duiven, The Nederlands). Mouse anti-human YKL-40 antibody (1:500, Quidel Corporation, San Diego, USA) was also tested for comparison purposes and to support specificity. Both antibodies showed similar staining patterns, though the former showed stronger YKL-40 immunoreactivity (Supplementary Fig. 1). After incubation with primary antibody, sections were washed with PBS and incubated for 30 min with biotin-conjugated rabbit anti-goat antibody at room temperature (1:400, DAKO, Glostrup, Denmark). Then sections were washed with PBS and a 60-min incubation with HRP-conjugated streptavidin antibody (1:300, DAKO) was performed. Sections were subsequently stained with 3,3-diaminobenzidine tetrahydrochloride dihydrate (DAB; 0.1 mg/mL, 0.02% H_2_O_2_, DAKO) for 10 min, rinsed in tap water and counterstained with hematoxylin for 1 min. After extensive washing in tap water, sections were dehydrated in a series of ethanol and xylene baths and mounted with Quick-D mounting medium (Klinipath, Duiven, The Nederlands). First, different dilutions of the primary and secondary antibodies were tested (Supplementary Fig. 2A-B). Thereafter, the specificity of the antibody for immunohistochemistry was tested by pre-absorption. The anti-Chitinase 3-like 1 antibody was pre-absorbed for 4 h at room temperature with 45 molar excess of recombinant protein (aa22–aa383, R&D), thereafter, sections were incubated with the pre-absorbed antibody overnight at 4°C (Supplementary Fig. 2C-D).

Positive or negative YKL-40 immunoreactivity was determined within either the temporal or frontal cortex region. Staining patterns were considered positive if YKL-40 immunoreactivity was present in two or more cell clusters within each section (Supplementary Fig. 3A). Staining patterns were considered negative if YKL-40 immunoreactivity was absent or present in only one cell per section (Supplementary Fig. 3B). In a subset of cases (temporal cortex from NDC = 6 and AD = 6), YKL-40 immunoreactivity was quantified by analyzing the DAB positive pixels with the QuPath software (thresholds: down-sample factor = 4, Gaussian sigma = 2, hematoxylin threshold (negative) = 0.1, DAB threshold (positive) = 0.3) as previously described [46]. Examination of the staining patterns was performed by a researcher who was blinded for diagnosis or other patient characteristics.

### Western blot

Human frontal cortex lysates (*n* = 104, 10 μg) were prepared in sample buffer (0.03 M Tris, 2% SDS, 10% glycerol, 50mM DTT, 0.1 mM bromophenol blue) and heated for 5 min at 95°C. Electrophoresis was performed using 1.5 mm NuPAGE Novex 4-12% Bis-Tris Protein Gels (Thermofisher Scientific, Waltham, USA) and proteins were transferred to polyvinylidene difluoride (PVDF) membranes (Millipore, Bedford, USA). Subsequently, PVDF membranes were blocked for 30 min with blocking buffer containing 5% (w/v) milk powder with 0.5% PBS Tween 20 (PBS-T) and incubated overnight at 4°C in blocking buffer containing either goat anti-human chitinase 3-like 1 antibody (1:200, R&D systems). After washing with wash buffer (PBS-T containing 0.05% (w/v) milk powder), PVDF membranes were incubated 1 h at room temperature with corresponding secondary antibodies (polyclonal rabbit anti-goat IgG/HRP (1:2000, DAKO, Glostrup, Denmark) or goat anti-mouse IgG/HRP (1:1000, DAKO)) in blocking buffer. Proteins were detected with the ECL^TM^ Western Blotting detection kit (GE Healthcare, Amersham, UK). After YKL-40 protein detection, PVDF membranes were incubated with stripping buffer (0.08M Tris-base, 2% SDS, 0.8% β-Mercaptoethanol; Sigma-Aldrich, Saint Louis, USA) for 30 min at 50°C to ensure removal of YKL-40 antibody. PVDF membranes were then re-incubated with mouse anti-human actin antibody (1:5000, clone AC-40, Sigma-Aldrich) following western blot procedure as described above. The protein signal was quantified using the ImageLab^TM^ software version 3.0 (Bio-Rad, Hercules, USA). A total of 8 blots were analyzed, all including a reference sample (frontal cortex) that was used to correct potential differences across blots (Supplementary Fig. 4). Actin reactivity was used as a protein loading control.

### Enzyme-linked immunosorbent assay

YKL-40 levels were measured in post-mortem brain lysates and CSF using MicroVue YKL-40 enzyme immunoassay (Quidel Corporation) following the manufacturer’s instructions. This kit was previously validated for analysis in CSF with intra- and inter-assay coefficient variations of < 4% and < 11% [27]. Additional analysis indicated that this kit could measure YKL-40 in post-mortem tissue with intra- and inter-assay variabilities < 8% and < 12%. Brain lysates and CSF samples were diluted 3-fold in sample dilution buffer and YKL-40 levels were corrected for dilution factor. Brain and CSF YKL-40 levels were normalized against the total protein content to analyze YKL-40 differences between pathological groups in brain lysates or between the paired CSF and brain samples.

### Statistical analysis

Statistical analysis was performed with IBM SPSS statistics (version 26, IBM, Armonk, NY). Normal distribution of the data was assessed using the Shapiro-Wilk test. Skewed data were normalized using Templeton’s two-step method if applicable [47]. The influence of different demographic variables on YKL-40 levels was determined by linear regression analysis, Student’s t-test or Mann–Whitney test. Differences in YKL-40 levels between groups were analyzed either by Pearson’s chi-square, Kruskal-Wallis test, or Analysis of covariance including center and age as covariates followed by Bonferonni post hoc analysis. YKL-40 levels in AD temporal and frontal cortex and FTLD frontal cortex were compared to controls from corresponding areas. Correlation between paired ante-mortem CSF and post-mortem brain was assessed using a Pearson correlation test. *p* values < 0.05 were considered significant.

## Results

### YKL-40 immunoreactivity and protein levels remain similar in post-mortem temporal and frontal cortex from AD and non-demented controls

**Fig. 1:**
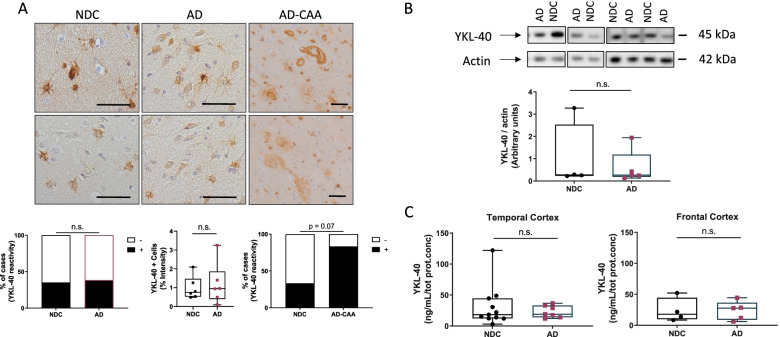
YKL-40 immunoreactivity and protein levels remain similar in post-mortem temporal and frontal cortex from AD and non-demented controls. **A** Representative images of paraffin sections from temporal cortex stained with the anti-YKL-40 antibody. YKL-40 immunoreactivity is present in glial and neuronal cells in NDC and AD cases. In AD-CAA sections, YKL-40 was detected around cerebral vessels and in structures resembling amyloid plaques. Scale bars represent 50μM. Semi-quantitation of YKL-40 immunoreactivity was performed by grouping cases into negative (i.e., zero or 1 positive cell) or positive (i.e., 2 or more positive cell groups). Stacked bar plots represent the percentage of cases with either negative (−, white area) or positive (+, black area) YKL-40 immunoreactivity in NDC (*n* = 51), AD (*n* = 52), and AD-CAA cases (*n* = 6). Box-dot plots show quantification of YKL-40 immunoreactivity by DAB+ pixel count in NDC (*n* = 6, Braak stage < I, Thal stage = 0) and AD (*n* = 6, Braak stage > V, Thal stage = 3) cases. **B** Representative immunoblot of YKL-40 in frontal cortex from NDC and AD patients. Actin was used as a loading control. Box-dot plot depict YKL-40 immunoblot reactivity corrected for actin protein loading in NDC (*n* = 4) and AD cases (*n* = 5). (C) YKL-40 levels were quantified by ELISA and corrected for total protein concentration in temporal cortex (NDC = 14 and AD = 6) and frontal cortex (NDC = 4 and AD = 5). Overall, no significant differences across groups were identified. Box represents median ± interquartile range with bars showing the lowest to highest points. Abbreviations: NDC, non-demented control; AD, Alzheimer’s disease; CAA, cerebral amyloid angiopathy; n.s., non-significant difference between groups

YKL-40 immunoreactivity was mainly present in glial cells but immunoreactivity was also detected in neuronal cells in the temporal cortex of both AD and controls (Fig. 1A). We observed that the number of YKL-40 positive cells was similar across groups (Fig. 1A). In a subset of cases, we also quantified the intensity of the YKL-40 positive immunoreactivity within a given cell and this did not differ either across pathological groups (Fig. 1A). We did not observe an association between YKL-40 immunoreactivity and the degree of Amyloid or Tau pathology as measured by Thal and Braak stages (Supplementary Fig. 5). Of note, strong YKL-40 immunoreactivity was detected around the cerebral vessels and in areas resembling amyloid plaques in AD cases with CAA pathology (AD-CAA, Fig. 1A), although differences did not reach statistical significance (NDC vs AD-CAA, *p* = 0.07, Fig. 1A). ELISA and western blot analysis indicate that the levels of brain YKL-40 in either the temporal or frontal cortex was similar between AD and controls (Fig. 1B, C). We confirmed that the ELISA employed could still detect differences in CSF YKL-40 concentration using a small CSF cohort of AD and controls (*p* = 0.015, Supplementary Fig. 6).

### YKL-40 immunoreactivity and protein levels remain similar in post-mortem frontal cortex from FTLD and non-demented controls

**Fig. 2:**
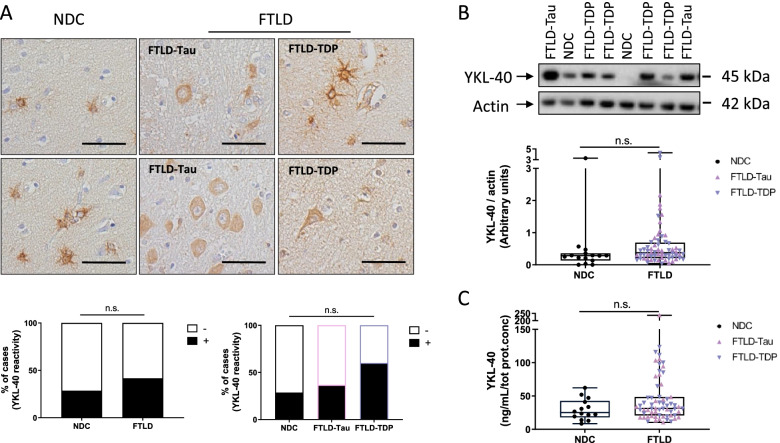
YKL-40 immunoreactivity and protein levels remain similar in post-mortem frontal cortex from FTLD and non-demented controls. **A** Representative images of paraffin sections from the frontal cortex stained with the anti-YKL-40 antibody. YKL-40 immunoreactivity is present in FTLD and control cases in glial and neuronal cells. Scale bars represent 50μM. Semi-quantitation of YKL-40 immunoreactivity was performed by grouping cases into either negative (i.e., zero or 1 positive cell) or positive (i.e., 2 or more positive cell groups). Stacked bar plots represent the percentage of cases with either negative (−, white area) or positive (+, black area) YKL-40 immunoreactivity in NDC (*n* = 7) and FTLD cases (*n* = 24; FTLD-Tau = 19 and FTLD-TDP = 5). **B** Representative immunoblot of YKL-40 in frontal cortex lysates. Actin was used as a loading control. Box-dot plots depict YKL-40 immunoblot reactivity corrected for actin in NDC (*n* = 14) and FTLD cases (*n* = 67; FTLD-Tau = 33, FTLD-TDP = 34). **C** YKL-40 levels were quantified by ELISA and corrected for total protein concentration in NDC (*n* = 14) and FTLD cases (*n* = 67; FTLD-Tau = 33 and FTLD-TDP = 34). Overall, no significant differences across groups were identified. Box represents median ± interquartile range with bars showing the lowest to highest points. Abbreviations: NDC, non-demented control; FTLD, frontal temporal lobar degeneration; n.s., non-significant difference between groups

In line with the findings described above, YKL-40 immunoreactivity was present in the frontal cortex from both FTLD cases and non-demented controls. We observed that YKL-40 immunoreactivity was mainly present in glial cells but it was also detected in neuronal cells (Fig. 2A). YKL-40 levels were similar between FTLD and non-demented controls as observed by either immunohistochemistry (Fig. 2A), western blot (Fig. 2B), or ELISA (Fig. 2C). No differences in brain YKL-40 levels were observed neither when the main FTLD pathological subtypes were analyzed separately (FTLD-Tau and FTLD-TDP; Fig. 2A–C) or between their sub-classifications (i.e., PSP, CBD, PiD, *MAPT*, *C9orf72*, *GRN*; Supplementary Fig. 7A-B).

### YKL-40 levels in ante-mortem CSF are higher and inversely associated with YKL-40 levels in post-mortem brain of dementia patients

**Fig. 3:**
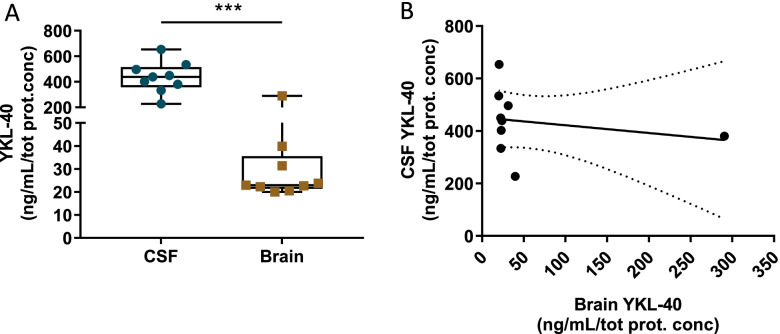
YKL-40 levels in ante-mortem CSF are higher and inversely associated with YKL-40 levels in post-mortem brain. **A** YKL-40 was quantified by ELISA and corrected for total protein concentration in paired ante-mortem CSF and post-mortem frontal cortex (*n* = 9). YKL-40 was 8-times higher in CSF compared to post-mortem frontal cortex (**B**). Scatter plot depicts a correlation between the two matrices; however, this did not reach significance (*r* = −0.49, *p* = 0.18). Box represents a median ± interquartile range with bars showing the lowest to highest points, *** *p* ≤ 0.001. Abbreviations: CSF, cerebrospinal fluid; tot prot. conc, total protein concentration

To further investigate the relationship between YKL-40 levels in brain and CSF, we analyzed the levels of YKL-40 in a small set of ante-mortem CSF samples paired with post-mortem frontal cortex from cases with FTLD and DLB pathology. The CSF YKL-40 concentration in this autopsy cohort ranged from 227 to 653 pg/mL, thereby covering the full range of CSF YKL-40 usually detected in other CSF studies. YKL-40 levels were 8-times higher in CSF compared to frontal cortex (*p* = 0.001, Fig. 3A). Though correlation data should be interpreted with caution considering the limited sample size, we observed an inverse, albeit non-significant, association between YKL-40 levels in the frontal cortex and the CSF (*r* = − 0.49, *p* = 0.18, Fig. 3B). A similar pattern was observed upon removing two outliers (*r* = − 0.448, *p* = 0.3, Supplementary Fig. 8).

## Discussion

In this study, we observed that the CSF YKL-40 changes detected in AD or FTD patients in this and previous studies were not detected in AD and FTLD pathological areas. We observed that YKL-40 levels are remarkably higher in CSF compared to brain. Interestingly, we found increased YKL-40 immunoreactivity in cases with AD-CAA pathology, suggesting a relationship of brain YKL-40 with CAA pathophysiology and/or vascular pathology.

CSF biomarker levels often reflect pathophysiological changes in the brain, as is the case for the classical AD CSF biomarkers: Aβ and hyperphosphorylated tau that reflect brain amyloidosis and tangle formation, respectively [48, 49]. However, AD is a multifaceted disorder in which multiple processes beyond amyloid and Tau are known to contribute to disease pathogenesis [50], including immunity [51]. Additional markers that reflect the neuroinflammatory changes underlying dementia might be useful not only to better define individual patients’ phenotype [52] but also to monitor treatment responses of anti-inflammatory drugs [11]. YKL-40 is involved in the immune system response [16, 33] and human neuropathological studies have shown an astrocytical YKL-40 immunoreactivity in cases with different neurological disorders [17, 22, 32, 34, 53]. Thus, we hypothesized that the elevated levels of YKL-40 in CSF of AD [17–24] and FTLD [20, 21, 23, 26–29] patients reflect neuroinflammatory changes in the brain areas that are typically affected in these dementia types. In agreement with previous studies, we observed that YKL-40 immunoreactivity was mainly found in clusters of glial cells (likely astrocytes) [17, 22, 32], and to some extent also in neurons. We observed that YKL-40 immunoreactivity was overall low with many cases (>50%) showing none or few YKL-40 positive cells. This is in line with a previous report showing that only 10% of all GFAP-astrocytes were positive for YKL-40 [32]. In agreement with a previous study, we also observed a prominent YKL-40 immunoreactivity around the cerebral vessels in AD cases with CAA pathology, suggesting a role of YKL-40 with vascular function [22]. However, while previous studies reported increased YKL-40 immunoreactivity in AD or FTLD cases [22, 32], we observed, using complementary methods, that the YKL-40 levels were similar in AD and FTLD compared to non-demented controls. Regarding AD, previous studies focused on frontal cortex areas while here we examined mainly the temporal cortex. The different regions analyzed could partly explain the discrepancies observed, especially considering that YKL-40 expression levels show some regional differences within the brain [22, 53]. Still, we did not observe any tendency in the small set of samples from AD frontal cortex. Noteworthy, our AD cohort (temporal cortex) is considerably large including more than 50 AD cases and controls, which increases the statistical power of our study. Regarding FTD, previous studies included only FTLD-Tau cases [32], while here we additionally included FTLD cases with TDP-43 pathology. However, we did not detect any differences when the FTLD pathological subtypes (i.e., FTLD-Tau and FTLD-TDP) were analyzed separately. It is unlikely that the observed immunohistochemically discrepancies are explained by the sample size as the FTLD group was comparable to previous studies. Considering the overall low expression pattern of YKL-40, the semi-quantitative methods applied in the different studies may partly explain the discrepancies across studies. We here analyzed the complete slide and dichotomized the outcomes into either positive or negative, and thus we might have missed subtle changes of YKL-40 immunoreactivity. Quantification of the intensity of YKL-40 in a subset of cases also showed similar results. Furthermore, we employed additional quantitative technologies, including the same immunoassay that is widely used for CSF analysis, which showed again no differences on YKL-40 levels between AD or FTLD and controls post-mortem brain. It is worth noting that a recent study showed no difference in *CHI3L1* mRNA post-mortem tissue from early-onset AD cases, which was attributed to the younger age of these patients [54]. In line with our findings, a recent mass-spectrometry-based study detected YKL-40 changes in the CSF of AD patients but not in post-mortem tissue [55]. Interestingly, using paired CSF-brain samples, we observed that CSF YKL-40 levels are 8-times higher than those detected in post-mortem frontal cortex. Strikingly, we observed a non-significant but moderate inverse association of YKL-40 between these two matrices but considering the low sample size of the paired samples (*n* = 9), these correlation findings should be interpreted with caution.

Our data thus overall suggest that the YKL-40 changes observed in CSF of dementia patients does not reflect changes of this neuroinflammatory protein in the brain areas that are typically affected. Interestingly, similar results have been observed previously for α-synuclein which was increased in CSF of CJD patients but not in post-mortem brain tissue [56]. CSF protein levels are dynamic and may change over time depending on the disease stage, as previously observed in longitudinal CSF biomarker studies performed in familial AD patients [57]. Thus, it could be possible that the YKL-40 changes observed in ante-mortem CSF in various reports might not be observed at the end stage of the disease within brain tissue. However, longitudinal studies have shown that CSF YKL-40 levels increased continuously with disease progression, suggesting that normalization of CSF YKL-40 levels with advancing stage may not explain the lack of differences in tissue [58, 59]. We neither observed an association of YKL-40 immunoreactivity with advanced pathological stages in brain tissue (i.e., Thal or Braak stages). The absence of YKL-40 changes in AD and FTLD post-mortem tissue together with the prominent reactivity of YKL-40 observed in AD with CAA pathology might indicate that CSF YKL-40 changes could be associated with the peripheral blood compartment. This is, however, less likely since YKL-40 levels in the blood are lower and do not correlate with those in CSF and its levels remain unchanged in the blood of dementia patients [27, 60]. YKL-40 is a protein secreted by various cell types including astrocytes [33, 61]. Thus, YKL-40 may quickly diffuse from cells to the extracellular space and the CSF, which may ultimately hamper its detection in post-mortem brain. The origin of the increased CSF YKL-40 levels consistently observed in AD and FTD patients is thus still not clear. CSF YKL-40 changes may originate from an alternative area not analyzed in the current study, such as the choroid plexus, which is involved in the production and regulation of CSF and has shown to express YKL-40 at least during brain development [62]. The choroid plexus could play an important role in facilitating the inflammatory process in the central nervous system. This is supported by others that show the presence of immune cells [63, 64] and an up-regulation of pro-inflammatory cytokines and chemokines in the choroid plexus of AD patients [65], suggesting an involvement of this area within the neuroinflammatory process associated with aging and AD [66, 67].

This study is not without limitations. The sample size of AD-CAA cases, as well as those with paired CSF-brain tissue, was small, and thus, such data should be confirmed in larger cohorts. In addition, our paired CSF-brain samples did not include AD patients or non-demented controls, and thus we can not exclude that the correlation of YKL-40 in brain and CSF might be different in these groups. However, the CSF YKL-40 values of the paired samples covered a wide concentration range (200–700 ng/mL) including YKL-40 values comparable to those previously detected in the control or AD groups. Furthermore, our study is limited to the frontal and temporal cortex, and thus other brain areas (e.g., choroid plexus) may contribute to the strong YKL-40 changes observed in CSF. We also acknowledge that we could not compare YKL-40 across different diseases due to the difference in brain regions. The strengths of our study are the use of complementary methods ensuring reliable measurements of YKL-40 protein and the high number of cases analyzed.

## Conclusion

The extensive analysis of YKL-40 performed here, using three different (semi-)quantitative technologies, suggests that the pathophysiological correlates underlying the increased CSF YKL-40 changes do not come from typically affected areas in AD or FTD. Understanding the origin of CSF YKL-40 changes is not trivial, considering its potential as a biomarker tracking ongoing neuroinflammation in different dementia types [10, 11, 68] or even as a novel potential therapeutic target [69, 70]. Interestingly, the prominent YKL-40 reactivity related to CAA pathology in the vessel wall suggests a potential involvement of brain YKL-40 levels in CAA pathophysiology which should be investigated in future studies.

## Supplementary Information

**Additional file 1: Supplementary Table 1**. Additional information for the individual cases included in this study.

**Additional file 2: Supplementary Table 2**. Demographic data of paired ante-mortem CSF and post-mortem tissue samples. **Supplementary Table 3**. Demographic details of CSF samples. **Supplementary figure 1**. Different antibodies show similar YKL-40 staining patterns. **Supplementary figure 2**. Antibody characterization. **Supplementary figure 3**. Representative image of the YKL-40 immunoreactivity semi-quantification. **Supplementary figure 4**. Full Western blot showing YKL-40 and actin protein bands in post-mortem frontal cortex from AD, FTLD and controls.

**Additional file 3: Supplementary figure 5**. YKL-40 immunoreactivity in post-mortem temporal cortex tissue does not correlate to pathology stages. **Supplementary figure 6**. YKL-40 levels are increased in CSF of AD patients. **Supplementary figure 7**. YKL-40 protein levels remain similar in post-mortem frontal cortex between the FTLD subclassifications and non-demented controls. **Supplementary figure 8**. YKL-40 levels in ante-mortem CSF are inversely associated with YKL-40 levels in post-mortem brain.

## Data Availability

The data that support the findings of this study are available from the corresponding author upon reasonable request.

## Abbreviations

AD: Alzheimer’s disease
CAA: Cerebral amyloid angiopathy
CBD: Corticobasal degeneration
CJD: Creutzfeldt-Jakob disease
DAB: Diaminobenzidine tetrahydrochloride dihydrate
DLB: Dementia with Lewy body
ELISA: Enzyme-linked immunosorbent assay
FTLD: Frontotemporal lobar degeneration
FTLD-ALS: FTLD with amyotrophic lateral sclerosis
MS: Multiple sclerosis
NDC: Non-demented controls
PiD: Pick’s disease
PSP: Progressive supranuclear palsy
PVDF: Polyvinylidene difluoride
T-PER: Tissue Protein Extraction Reagent
