# Supplementary material for: YKL-40 changes are not detected in post-mortem brain of patients with Alzheimer’s disease and frontotemporal lobar degeneration
Source: Alzheimers Res Ther. 2022 Jul 25;14:100. doi: 10.1186/s13195-022-01039-y (PMC9310415; doi:10.1186/s13195-022-01039-y)
Supplement: Supplementary file 3 — Additional file 3: Supplementary figure 5. YKL-40 immunoreactivity in post-mortem temporal cortex tissue does not correlate to pathology stages. Supplementary figure 6. YKL-40 levels are increased in CSF of AD patients. Supplementary figure 7. YKL-40 protein levels remain similar in post-mortem frontal cortex between the FTLD subclassifications and non-demented controls. Supplementary figure 8. YKL-40 levels in ante-mortem CSF are inversely associated with YKL-40 levels in post-mortem brain. [file 13195_2022_1039_MOESM3_ESM.docx]

## **Additional File 3**

Supplemental Table 1. Demographic details of CSF samples

A

B

Supplementary figure 5. YKL-40 immunoreactivity in post-mortem temporal cortex tissue does not correlate to pathology stages.

Semi-quantitation of YKL-40 immunoreactivity was performed by grouping cases into negative (i.e. zero or 1 positive cell) or positive (i.e. 2 or more positive cell groups). Stacked bar plots represent the percentage of cases with either negative (-, white area) or positive (+, black area) YKL-40 immunoreactivity in NDC and AD cases temporal cortex were grouped according to Thal (A) or Braak (B) stages. A total of 103 individuals were analyzed for Thal stages including individuals with stage 0 (n=26), stage 1 (n=10), stage 2 (n=18) and stage 3 (n=49). A total of 103 individuals were analyzed for the braak stages including braak stage 0 (n=9), stage I (n=25), stage II (n=14), stage III (n=10), stage IV (n=12), stage V (n=15) and stage VI (n=18). We did not observe a significant trend between YKL-40 immunoreactivity and either Thal or Braak stages.

Supplementary figure 6. YKL-40 levels are increased in CSF of AD patients.

YKL-40 was quantified by ELISA in ante-mortem CSF of controls (n = 13) and patients with AD (n = 45). We found that YKL-40 levels were increased in AD patients compared to controls. Box represent median ± interquartile range with bars showing the lowest to highest points, ** P ≤ 0.01, abbreviations: CON, controls; AD, Alzheimer's Disease; CSF, cerebrospinal fluid

A

B

Supplementary figure 7. YKL-40 protein levels remain similar in post-mortem frontal cortex between the FTLD subclassifications and non-demented controls.

(A) Box-dot plot depict YKL-40 immunoblot reactivity corrected for actin in NDC (n = 14) and the FTLD subclassifications cases (FTLD-Tau includes: PSP = 12, CBD = 3, PiD = 3, *MAPT* = 15 and FTLD-TDP includes: TDP pathology = 21, *C9orf72* = 7, *GRN* = 6)). (B) YKL-40 levels were quantified by ELISA and corrected for total protein concentration in the same cases. Overall, no significant differences across groups were identified and no trend became apparant. Box represents median ± interquartile range with bars showing the lowest to highest points. Abbreviations: NDC, non-demented control; FTLD, frontal temporal lobar degeneration; n.s. non-significant difference between groups.

Supplementary figure 8. YKL-40 levels in ante-mortem CSF are inversely associated with YKL-40 levels in post-mortem brain.

YKL-40 was quantified by ELISA and corrected for total protein concentration in paired ante-mortem CSF and post-mortem frontal cortex (n = 7). The scatter plot depict a correlation between the two matrices with two outliers removed, however, this did not reach significance (*r* = -0.448, p = 0.3). Abbreviations: CSF, cerebrospinal fluid; tot prot. conc, total protein concentration.
