# Supplementary material for: YKL-40 changes are not detected in post-mortem brain of patients with Alzheimer’s disease and frontotemporal lobar degeneration
Source: Alzheimers Res Ther. 2022 Jul 25;14:100. doi: 10.1186/s13195-022-01039-y (PMC9310415; doi:10.1186/s13195-022-01039-y)
Supplement: Supplementary file 2 — Additional file 2: Supplementary Table 2. Demographic data of paired ante-mortem CSF and post-mortem tissue samples. Supplementary Table 3. Demographic details of CSF samples. Supplementary figure 1. Different antibodies show similar YKL-40 staining patterns. Supplementary figure 2. Antibody characterization. Supplementary figure 3. Representative image of the YKL-40 immunoreactivity semi-quantification. Supplementary figure 4. Full Western blot showing YKL-40 and actin protein bands in post-mortem frontal cortex from AD, FTLD and controls. [file 13195_2022_1039_MOESM2_ESM.docx]

## **Additional File 2**

Supplemental Table 1. Demographic details of CSF samples

### Material and methods

#### CSF samples

CSF samples (n = 69) were selected from the Amsterdam Dementia Cohort (1, 2), which included patients with clinical diagnosis of AD dementia (n = 45) and controls (cases with subjective memory complaints, n = 13). All participants underwent standard neurological and cognitive assessments (2) and the diagnosis was assigned according to international consensus criteria for AD (3). AD CSF biomarker profile (CSF total tau (t-Tau)/Aβ42 ratio > 0.52) was used to support AD diagnoses (4). Individuals who presented with cognitive complaints but performed normally on clinical investigations (i.e., criteria for MCI, dementia, or any psychiatric disorder were not met) served as controls. These controls were additionally selected based on a negative AD biomarker profile (4). CSF was collected by standard lumbar puncture and stored according to consensus guidelines (5). Informed consent was provided from subjects or their next of kin. Patient clinical details are presented in supplementary table 2.

Supplementary Table 2. Demographic data of paired ante-mortem CSF and post-mortem tissue samples

|  | Paired CSF – Post mortem tissue |
| --- | --- |
| n (M/F) | 9 (5/4) |
| PMD, hours (mean ± SD) | 13.9 (7.5) |
| time interval CSF and post-mortem collection, years (mean ± SD) | 1.4 (1.8) |
| Diagnosis | 5 FTLD-TDP |
|  | 3 FTLD-Tau |
|  | 1 DLB |

FTLD, frontotemporal lobar degeneration; TDP, TAR DNA-binding protein; DLB, dementia with lewy body; n, number of cases; M, Male; F, female; PMD, post-mortem delay; CSF, cerebral spinal fluid

Supplementary Table 3. Demographic details of CSF samples

|  | CON | AD |
| --- | --- | --- |
| n (M/F) | 13 (5/8) | 45 (20/25) |
| Age, years | 66 (7) | 68 (7) |
| MMSE score | 29 (1) | 20 (5)*** |
| CSF Aβ1-42 (pg/mL) | 980 (201) | 444 (125)*** |
| CSF t-Tau (pg/mL) | 261 (98) | 675 (380)*** |
| CSF p-Tau (pg/mL) | 49 (13) | 99 (47)*** |

Age, MMSE score and cerebrospinal fluid biomarker levels are presented as mean ± standard deviation.

CON, controls with subjective cognitive decline; AD, Alzheimer’s disease; n, number of cases; M, Male; F, female; MMSE, Mini-Mental State Examination; CSF, cerebrospinal fluid; t-Tau, total tau; p-Tau, phosphorylated tau

*** indicate p < 0.001 compared to CON

### Results

A

B

C

D

Supplementary figure 1. Different antibodies show similar YKL-40 staining patterns.

For immunohistochemistry analysis, first two different YKL-40 antibodies were tested. The Goat anti-human Chitinase 3-like 1 Antibody (R&D Systems, A-B) and the Mouse anti-human YKL-40 antibody (Quidel Corporation, C-D). We observed similar staining patterns between the two antibodies though the Goat anti-human Chitinase 3-like 1 Antibody (R&D Systems) showed the strongest staining (A-B). Scale bars in A-C represent 100µM and scale bars in B-D represent 50µM.

A

B

C

D

Supplementary figure 2. Antibody characterization.

For immunohistochemistry analysis, first two different dilutions of the anti-YKL-40 antibody in combination with HRP-conjugated streptavidin antibody (streptavidin-HRP) were tested. Dilution 1: 1:50 YKL-40 antibody with 1:300 streptavidin-HRP (A) and dilution 2: 1:100 YKL-40 antibody with 1:100 streptavidin-HRP (B). YKL-40 immunoreactivity in post-mortem temporal cortex show reactivity in neuronal and glial cells, which decreased with higher antibody dilutions (A-C) and signal was abolished after the antibody was pre-absorped with YKL-40 recombinant protein (D). Scale bars represent in A-B represent 100µM and scale bars in C-D represent 50µM.

A

B

Supplementary figure 3. Representative image of the YKL-40 immunoreactivity semi-quantification.

Two temporal cortex sections from AD cases are representered were semi-quantitation of YKL-40 immunoreactivity was performed by grouping cases into positive (i.e. 2 or more positive cell groups, A) or negative (i.e. zero or 1 positive cell, B). Scale bars represent represent 100µM.

B

Supplementary figure 4. Full Western blot showing YKL-40 and actin protein bands in post-mortem frontal cortex from AD, FTLD and controls.

(A) Full length western blots depict YKL-40 (top panel) and Actin (bottom panel) protein bands from NDC, AD and FTLD frontal cortex lysates corresponding to the expected molecular weight of the proteins 45 kDa and 42 KDa, respectively. X indicate samples from different diagnosis that were not included in the analysis or an empty well. (B) Dot plot depict YKL-40 immunoblot reactivity corrected for actin protein and the reference sample in frontal cortex lysates. Abbreviations: NDC, non-demented control; AD, Alzheimer’s disease; FTLD, Frontal temporal lobar degeneration.

### References

1.	van der Flier WM, Scheltens P. Amsterdam Dementia Cohort: Performing Research to Optimize Care. J Alzheimers Dis. 2018;62(3):1091-111.

2.	van der Flier WM, Pijnenburg YA, Prins N, Lemstra AW, Bouwman FH, Teunissen CE, et al. Optimizing patient care and research: the Amsterdam Dementia Cohort. J Alzheimers Dis. 2014;41(1):313-27.

3.	McKhann GM, Knopman DS, Chertkow H, Hyman BT, Jack CR, Jr., Kawas CH, et al. The diagnosis of dementia due to Alzheimer's disease: recommendations from the National Institute on Aging-Alzheimer's Association workgroups on diagnostic guidelines for Alzheimer's disease. Alzheimers Dement. 2011;7(3):263-9.

4.	Duits FH, Teunissen CE, Bouwman FH, Visser PJ, Mattsson N, Zetterberg H, et al. The cerebrospinal fluid "Alzheimer profile": easily said, but what does it mean? Alzheimers Dement. 2014;10(6):713-23 e2.

5.	Teunissen CE, Petzold A, Bennett JL, Berven FS, Brundin L, Comabella M, et al. A consensus protocol for the standardizarion of CSF collection and biobank. Neurology. 2009;73:1914-22.
